# Chicago Medical Society

**Published:** 1879-05

**Authors:** 


### ARTICLE VII.

CHICAGO MEDICAL SOCIETY, Regular Meeting, being the Twenty-seventh Annual Meeting, April 7th, 1879. Dr. Ingals, president, in the chair.

The Secretary, Dr. Graham, read his report, which while it showed one hundred and eighty-five members at present on the society's roll, did not, he said, represent more than one-half the profession of the city eligible for membership.

The result of the election for new officers was as follows:

President, Dr. E. Andrews; Vice-President, Dr. R. G. Bogue; Secretary, Dr. D. W. Graham; Treasurer, Dr. F. H. Davis.

An honorarium was tendered to the secretary for his efficient services during the past year, and one to the proprietor of the Grand Pacific Hotel, Mr. Drake, for courtesies extended to the society in providing commodious quarters for their meetings.

A committee was appointed to see the new Mayor-elect, and desire him to separate the duties of the sanitary officials from the domination of politics, and to retain in office the present Health Commissioner, Dr. De Wolf.

After the customary brief addresses from the retiring and the newly elected officers, the society adjourned.

